# LncRNA *MT1JP* functions as a ceRNA in regulating FBXW7 through competitively binding to miR-92a-3p in gastric cancer

**DOI:** 10.1186/s12943-018-0829-6

**Published:** 2018-05-02

**Authors:** Gang Zhang, Shuwei Li, Jiafei Lu, Yuqiu Ge, Qiaoyan Wang, Gaoxiang Ma, Qinghong Zhao, Dongdong Wu, Weida Gong, Mulong Du, Haiyan Chu, Meilin Wang, Aihua Zhang, Zhengdong Zhang

**Affiliations:** 1 Department of Neurology, Children’s Hospital of Nanjing Medical University, Nanjing, China; 2 Department of Environmental Genomics, School of Public Health, Jiangsu Key Laboratory of Cancer Biomarkers, Prevention and Treatment, Collaborative Innovation Center for Cancer Personalized Medicine, Nanjing Medical University, 101 Longmian Avenue, Jiangning District, Nanjing, 211166 China; 3 Department of Genetic Toxicology, The Key Laboratory of Modern Toxicology of Ministry of Education, School of Public Health, Nanjing Medical University, Nanjing, China; 4 Department of General Surgery, The Second Affiliated Hospital of Nanjing Medical University, Nanjing, China; 5 Department of General Surgery, Yixing Cancer Hospital, Yixing, China; 6 Key Laboratory of Environmental Pollution Monitoring and Disease Control, Ministry of Education, Guizhou Medical University, Guiyang, 550025 Guizhou China

**Keywords:** lncRNA, *MT1JP*, Gastric cancer, ceRNA, Prognosis

## Abstract

**Background:**

Emerging evidence has shown that dysregulation function of long non-coding RNAs (lncRNAs) implicated in gastric cancer (GC). However, the role of the differentially expressed lncRNAs in GC has not fully explained.

**Methods:**

LncRNA expression profiles were determined by lncRNA microarray in five pairs of normal and GC tissues, further validated in another 75 paired tissues by quantitative real-time PCR (qRT-PCR). Overexpression of lncRNA *MT1JP* was conducted to assess the effect of *MT1JP* in vitro and in vivo. The biological functions were demonstrated by luciferase reporter assay, western blotting and rescue experiments.

**Results:**

LncRNA *MT1JP* was significantly lower in GC tissues than adjacent normal tissues, and higher *MT1JP* was remarkably related to lymph node metastasis and advance stage. Besides, GC patients with higher *MT1JP* expression had a well survival. Functionally, overexpression of lncRNA *MT1JP* inhibited cell proliferation, migration, invasion and promoted cell apoptosis in vitro, and inhibited tumor growth and metastasis in vivo. Functional analysis showed that lncRNA *MT1JP* regulated FBXW7 expression by competitively binding to miR-92a-3p. MiR-92a-3p and down-regulated FBXW7 reversed cell phenotypes caused by lncRNA *MT1JP* by rescue analysis.

**Conclusion:**

*MT1JP*, a down-regulated lncRNA in GC, was associated with malignant tumor phenotypes and survival of GC. *MT1JP* regulated the progression of GC by functioning as a competing endogenous RNA (ceRNA) to competitively bind to miR-92a-3p and regulate FBXW7 expression. Our study provided new insight into the post-transcriptional regulation mechanism of lncRNA *MT1JP*, and suggested that *MT1JP* may act as a potential therapeutic target and prognosis biomarker for GC.

**Electronic supplementary material:**

The online version of this article (10.1186/s12943-018-0829-6) contains supplementary material, which is available to authorized users.

## Background

Recently, high-throughput genome and transcriptome sequencing and microarrays have indicated that apart from protein-coding genes, 75% of the human genomes is transcribed into noncoding RNAs [1, 2]. LncRNAs are functionally catalogued as noncoding transcripts are more than 200 nucleotides in length, and have no potential protein-coding ability. The Encyclopedia of DNA Elements (ENCODE) Project Consortium revealed that more than 28,000 lncRNAs were transcribed in the whole genomes [2]. The aberrant expression and deficiency or mutation of lncRNAs were reported to be involved in numerous complex diseases, including cancers [3, 4]. Mounting evidence indicated that lncRNAs are implicated in a variety of biological processes, including chromatin interaction, transcription regulation, mRNA post-transcriptional regulation and epigenetic regulation [5–7].

Additionally, increasing experimental evidence supports that lncRNA functions as competitive endogenous RNA (ceRNA), which compete for microRNA (miRNA) to up-regulate the expression of a target gene. The ceRNA hypothesis provide new insights into the function of a large amount of uncharacterized lncRNAs [8]. It has been reported that muscle-specific long noncoding RNA (linc-MD1) regulate MAML1 and MEF2C expression by sponging miR-133 and miR-135 [9]. Another study has shown that lncRNA BC032469 acted as a ceRNA for miR-1207-5p to up-regulate the expression of hTERT and promoted proliferation in gastric cancer (GC) [10].

GC is the fourth most frequent malignancy and contributes to the second leading cause of cancer mortality. Although effective medical treatments such as surgery, chemotherapy and radiation have been improved, GC patients are usually diagnosed with advanced stage, resulting in a low five-year survival rate [11–13]. Currently, GC is still a globe health problem, which highlights the need for further studies of molecular mechanism of GC and identify effective therapeutic targets. Emerging evidence have shown that aberrant expression of many lncRNAs were observed in gastric cancer and significantly associated with carcinogenesis, diagnosis and prognosis of gastric cancer [14, 15]. However, the role of lncRNA and its molecular mechanism involved in GC remain largely obscure. To systematically identify lncRNAs involved in the carcinogenesis of GC, we analyzed and integrated the results of our lncRNA microarray and Gene Expression Omnibus (GEO) database. Among the deregulated lncRNAs, we selected and investigated *MT1JP*, a lncRNA located at 16q12.2 region. Here, we found that lncRNA *MT1JP* acted as a competing endogenous RNA in regulating FBXW7 through sponging miR-92a-3p and inhibit cell proliferation, migration, invasion and promote cell apoptosis.

## Methods

### GC tissues

A total of 80 pairs of matched normal and GC tissues were collected from The Second Affiliated Hospital of Nanjing Medical University between February 2009 and October 2013. Five paired adjacent normal tissues and GC tissues were randomly selected in lncRNA microarrays study, and the remaining 75 paired gastric tissues were applied to qRT-PCR analysis. Additionally, another 330 paraffin-embedded GC tissues and corresponding follow-up information were obtained from Nantong Tumor Hospital between February 2008 and March 2013. All subjects have written informed consent and this study was approved by the Institutional Review Boards of Nanjing Medical University.

### LncRNA microarrays

The lncRNA expression characteristics of GC were investigated by Arraystar Human LncRNA microarray V2.0, which contains 30,215 coding genes and 33,045 lncRNAs collected from several databases such as UCSC, Ensembl, RefSeq and the lncRNAs reported from literatures were also included. The microarray and data collection were conducted by KangChen Bio-tech (Shanghai, PR China). The details are as mentioned previously [16]. In addition, non-coding RNA profiling GSE53137 from the same platform was downloaded from GEO database, which investigate lncRNAs expression in six pairs of human gastric adenocarcinoma and adjacent normal tissues. Paired *t*-test was conducted to assess the differentially expressed lncRNAs between tumor and adjacent normal tissues (fold change > 2.0 and *P* value < 0.05).

### qRT-PCR analysis

The total RNA from GC tissue or cell lines were extracted using Trizol Reagent (Invitrogen, CA, USA) and mirVana miRNA Isolation Kit (Applied Biosystems) according to the manufacturer’s instructions. M-MLV reverse transcriptase (Invitrogen) was used for lncRNA *MT1JP* reverse transcription. The expression of lncRNA *MT1JP* and FBXW7 was detected by ABI 7900HT Real-Time PCR System (Applied Biosystem, Foster City, CA, USA), using SYBR Green assays (TaKaRa Biotechnology, Dalian, China) and GAPDH was used as the internal control. The expression of miR-92a-3p was measured using TaqMan MicroRNA Assays (Applied Biosystems) and U6 was treated as an internal control. All the primer sequences were available in Additional file 1: Table S2.

### LncRNA coding capacity prediction

Coding Potential Assessment Tool (CPAT, http://lilab.research.bcm.edu/cpat/) was used to assess the coding capacity of lncRNA *MT1JP*. The CPAT conducted a logistic regression model by using the sequence features of open reading frame coverage, open reading frame size, hexamer usage bias and Fickett TESTCODE statistic. The CPAT chose 0.364 as a cutoff as human coding probability (CP). CP < 0.364 suggests noncoding sequence, whereas CP ≥ 0.364 indicates coding sequence [17].

### Nuclear-cytoplasmic fractionation

Nuclear/cytoplasmic fractionation was conducted by the Protein and RNA Isolation System (Ambion) according to the manufacturer’s protocols. U6 was treated as a nuclear control while GAPDH was a cytoplasmic control.

### Cell proliferation, migration, invasion, apoptosis and cell cycle analysis

The Cell Counting Kit 8 (Dojindo) was used to measure cell viability. The spectrophotometric absorbance at 450 nm for each sample was detected using spectrophotometer Infinite M200 (Tecan). All the experiments were repeated three times in six replicates. The transwell assay was used to evaluate cell migration. Cell invasion was assessed using BioCoat Matrigel Invasion Chamber (BD Biosciences Discovery Labware). Cell numbers for cell migration and invasion in three random fields were counted. Cells were stained by Annexin V and propidium iodide using the Annexin V–FITC Apoptosis Detection kit (Invitrogen), and the percentage of apoptosis was examined with flow cytometry (BD Bioscience, San Jose, CA, USA). For detection of cell cycle, cells were stained with PI after 48 h transfection and examination were performed by FACS Calibur system (Beckman Coulter).

### Luciferase reporter assay

The full-length lncRNA *MT1JP* cDNA was cloned into the BamHI and XhoI enzyme restriction sites of psiCHECK-2 vector (Promega) (psicheck-2-*MT1JP*-wild vector). The potential miR-92a-3p binding sites were mutated by the QuikChang site-directed mutagenesis kit (Agilent Technologies) (psicheck-2-*MT1JP*-mut vector). The psicheck-2-*MT1JP*-wild vector or psicheck-2-*MT1JP*-mut vector and miR-92a-3p mimics were co-transfected into BGC-823 and SGC-7901 cell by Lipofectamine 2000 (Invitrogen, Carlsbad, CA, USA). The wild and mutant miR-92a-3p was cloned into the BamHI and XhoI enzyme restriction sites of psiCHECK-2 vector (Promega). miR-92a-3p-wild or miR-92a-3p-mutant and lncRNA *MT1JP* overexpression vector were co-transfected into both SGC-7901 and BGC-823 cell by Lipofectamine 2000 (Invitrogen, Carlsbad, CA, USA). The luciferase activity was assessed by Dual-Luciferase Reporter Assay System (Promega, Madison, WI, USA) and the firefly luciferase activity was normalized by renilla luciferase activity.

All the cloned sequences were validated by DNA sequencing.

### Tumor formation test

Twenty (10 nude mice in each group) five-week-old female athymic BALB/c nude mice were kept under pathogen-free conditions. BGC-823 cells that were transfected with an empty vector and the MT1JP vector were collected and resuspended at a concentration of 1.5 × 10^7^ cells/mL. Then, 0.1 mL of the cells that were transfected with the MT1JP vector and an empty vector were subcutaneously injected into each posterior flank of the nude mice. The tumor weights and volumes were examined every 3 days. The mice were killed after 16 days post-injection, and the tumors from the mice were measured.

### Immunohistochemistry (IHC)

Hematoxylin and eosin (H&E) staining was applied to select representative areas. The antibodies against Ki-67 (Abcam, Cambridge, MA, USA) were applied for IHC. The process of IHC was according to our previous study [18].

### Western blotting analysis

Protein was isolated from BGC-823 and SGC-7901 cell lines as previously stated [19].

Forty micrograms of total protein s were run on a 14.7% polyacrylamide gel, and then transferred to polyvinylidene difluoride membranes (Hybondenhanced chemiluminescence; Amersham Pharmacia Biotech). The antibodies against FBWX7(1:1000) and β-actin (1:1000) were purchased from Abcam (Abcam, Cambridge, MA, USA). The antibodies against Caspase-9 antibody (1:1000) and Caspase-3 (1:1000) were purchased from Cell Signaling Technology (Cell Signaling Technology, USA). The protein was measured with a Phototope–horseradish peroxidase Western blot detection kit (Cell Signaling Technology, Inc.), and β-actin was treated as an internal control.

### Statistical analysis

For continuous variables, the results were shown as mean ± SD. Student’s *t*-test was applied to compare the difference of means between two groups. Differentially expressed *MT1JP* between gastric cancer and normal tissues from TCGA database was also evaluated by Student’s *t*-test. We used Kaplan-Meier curve and log-rank test to evaluate the effect of lncRNA *MT1JP* on survival of GC patients. The relationship between lncRNA *MT1JP* and *FBXW7* expression was assessed by Spearman Pearson correlation analysis. A two-sided *P* value < 0.05 was considered as statistically significant. All analyses were performed using SAS software (version 9.2; SAS Institute, Inc., Cary, NC, USA).

## Results

### Differently expressed lncRNA identified in microarray and GEO database

LncRNA microarray was conducted in five pairs of GC tissues and adjacent normal tissues to investigate the lncRNA expression signatures of GC. Compared with normal tissues, 551 lncRNAs were up-regulated and 278 lncRNAs were down-regulated in GC tissues (fold change > 2.0 and *P* < 0.05). In order to reduce false positive, GSE53137, lncRNA profile based on the same microarray platform as our study, was download from GEO database. We integrated and selected the consistently differently expressed lncRNAs in both GSE53137 database and our microarray. The top 10 significantly up-regulated and down-regulated candidate lncRNAs are shown in Additional file 1: Table S1. Given that among the differently expressed lncRNAs, the fold change of *MT1JP* was the most remarkable, therefore, we focused on lncRNA *MT1JP* in the further study.

### Characterization of lncRNA *MT1JP* in GC

**Fig. 1:**
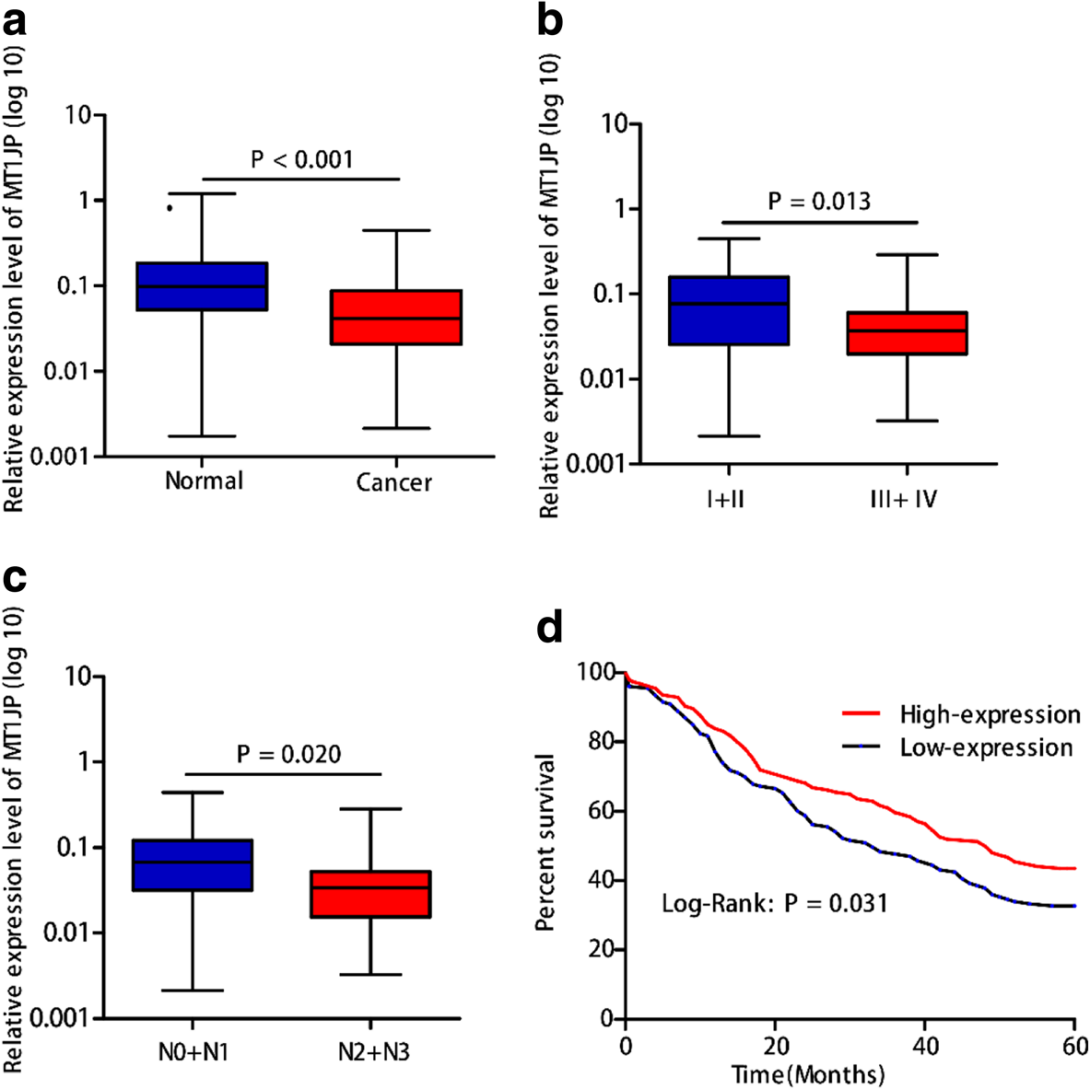
LncRNA *MT1JP* was significantly up-regulated in GC tissue and associated with progression and prognosis of GC. **a** Relative expression level of lncRNA *MT1JP* in GC tissue and adjacent normal tissues. **b** Relative expression level of lncRNA *MT1JP* in GC patient with early stage (I/II) and advanced stage (III/IV). **c** Differential expression levels of lncRNA between GC patients with or without lymph node metastasis. **d** Kaplan-Meier curve showing survival in GC patients stratified by lncRNA *MT1JP* expression. Patients were divided into high (red) and low expression group (blue) according to the median of lncRNA *MT1JP* expression

Subsequently, the expression levels of lncRNA *MT1JP* were explored in a large-cohort of 75 pairs of GC tissues and corresponding adjacent normal tissues. Significantly lower *MT1JP* expression was observed in GC tissues than in adjacent normal tissues (*P* < 0.001, Fig. 1a).Similar result was also acquired from The Cancer Genome Atlas (TCGA) database (*P* = 0.018, Additional file 1: Figure S1). We then examined the clinic pathological role of lncRNA *MT1JP*, and found that higher *MT1JP* expression was significantly related to lymph node metastasis and advance stage (*P* = 0.020 and 0.013, respectively, Fig. 1b and c). We further investigate the association between lncRNA *MT1JP* and survival of 308 GC patients with follow-up data. Kaplan-Meier curve and log-rank results indicated that GC patients with higher lncRNA *MT1JP* expression had a well prognosis and longer survival (HR = 1.33, 95%CI = 1.02–1.76, log-rank *P* = 0.031, Fig. 1d).

### LncRNA *MTIJP* overexpression inhibited cell proliferation, migration and invasion and promoted cell apoptosis in vitro

Consider that lncRNA *MT1JP* was down-regulated in GC tissues, we next investigated the effects of lncRNA *MT1JP* overexpression on GC cell phenotypes.

**Fig. 2:**
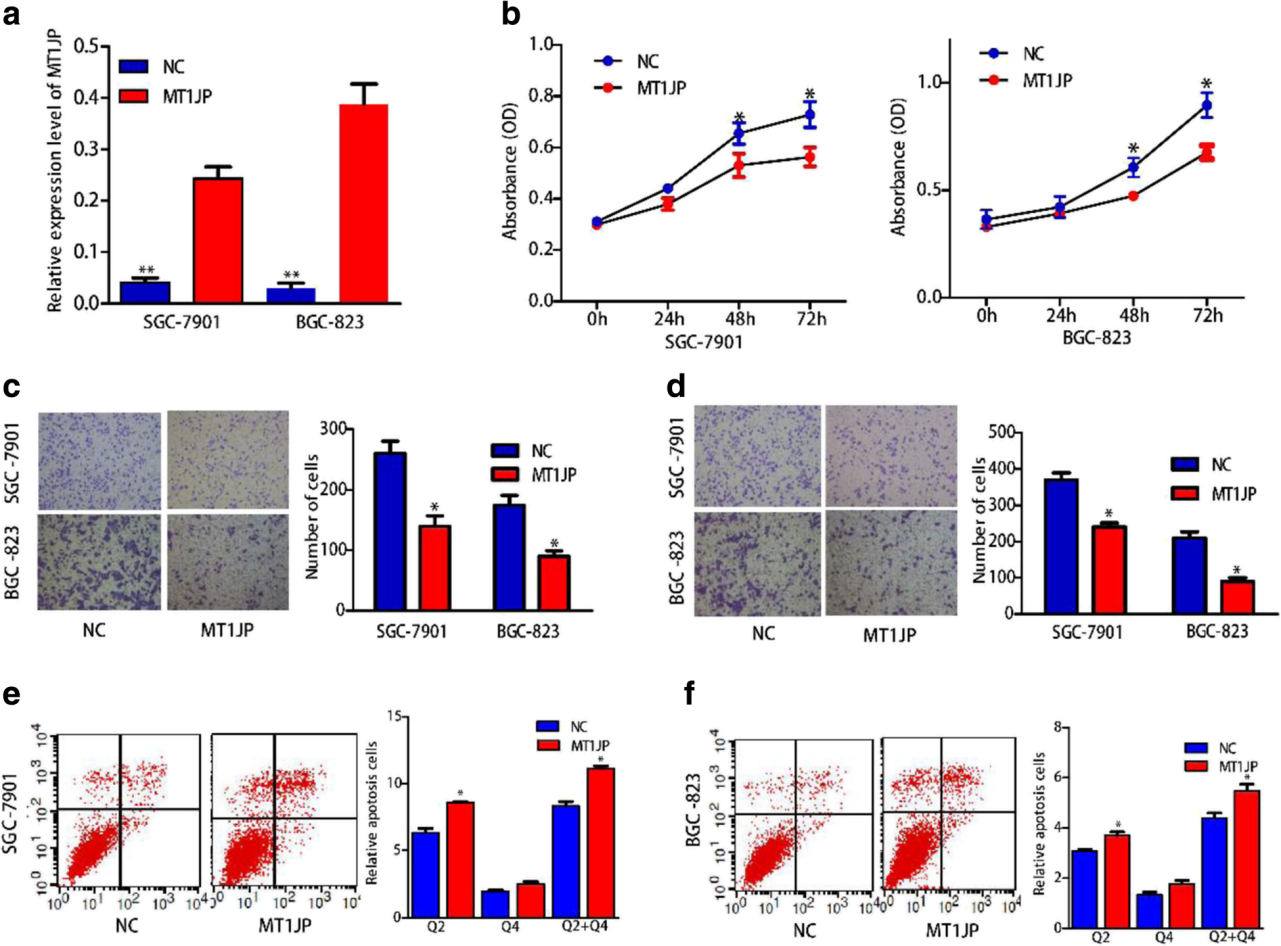
LncRNA *MT1JP* inhibited GC cell proliferation, migration, invasion and promoted cell apoptosis. **a** Effect of lncRNA *MT1JP* overexpression on proliferation of GC cell lines. **b**, **c** Overexprssion of lncRNA *MT1JP* significantly inhibited migration and invasionof GC cell. **d** Overexprssion of lncRNA *MT1JP* significantly enhancedapoptosis of GC cell. *MT1JP* induces the apoptosis of SGC7901 (**e**) and MGC-823 (**f**)

Because the expression of *MT1JP* was relatively lower in SGC-7901 and BGC-823 cells, we performed overexpression experiments in these two cell lines (Additional file 1: Figure S1B). Constructed pEGFP-N1 vector containing full-length lncRNA *MT1JP* was transfected into SGC-7901cell and BGC-823 cell, and found that the expression was effectively up-regulated as compare with negative control (NC) (Fig. 2a). Intriguingly, lncRNA *MT1JP* overexpression markedly inhibit cell proliferation in both SGC-7901and BGC-823 cells (Fig. 2b). Moreover, cell migration and invasion abilities in both GC cell lines were also significantly suppressed by lncRNA *MT1JP* overexpression (Fig. 2c and d). Flow cytometry analysis demonstrated that compared with NC, lncRNA *MT1JP* overexpression prominently promoted cell apoptosis in GC cells (Fig. 2e and f).

### LncRNA *MT1JP* overexpression inhibited tumor growth and metastasis in vivo

**Fig. 3:**
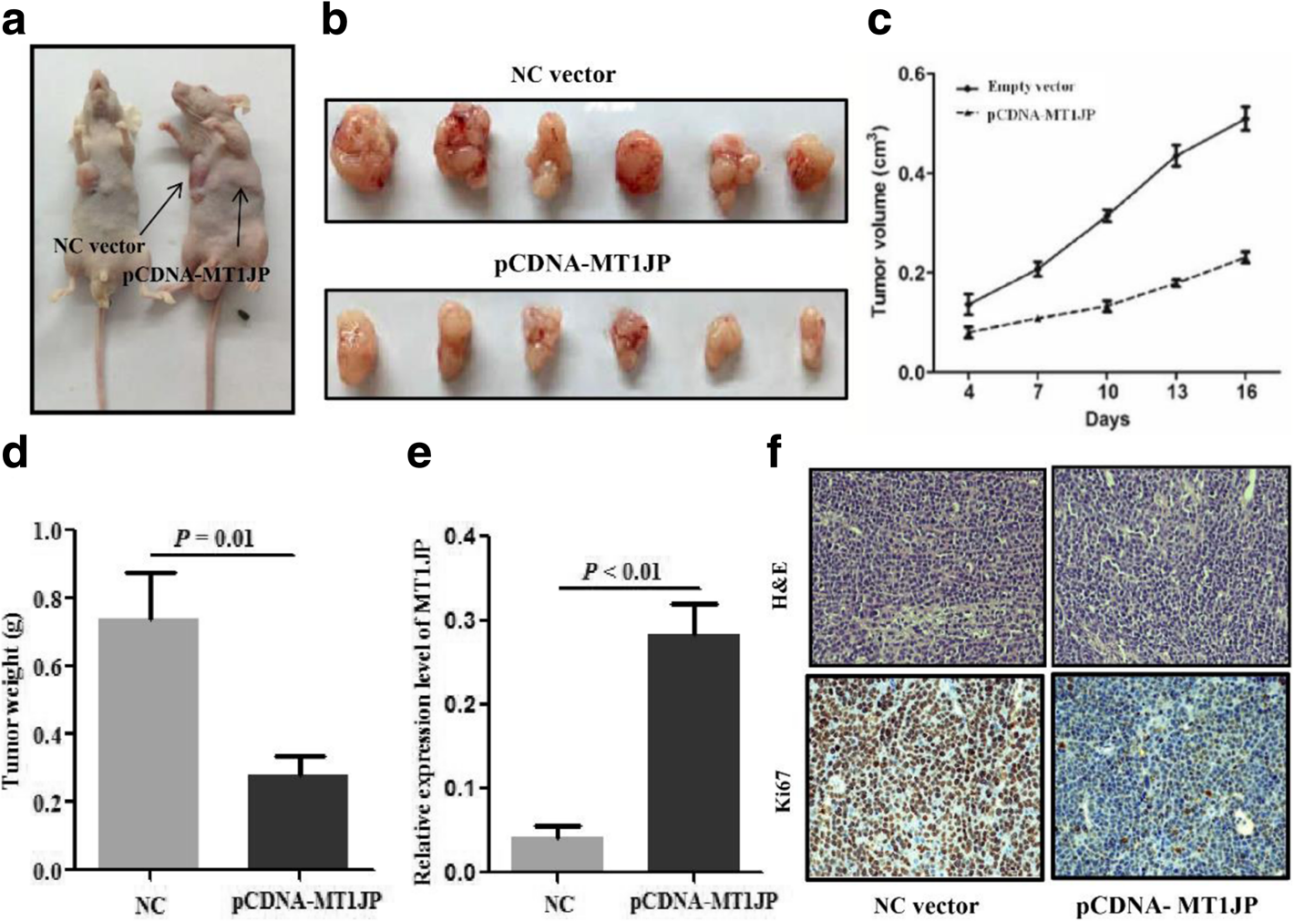
Xenograft tumor formation of GC cell with overexpression of lncRNA *MT1JP* in nude mice. **a**-**d** Overexpression of lncRNA *MT1JP* inhibited the growth of transplanted BGC-823 GC cell in nude mice. **e** Relative expression level of *MT1JP* of xenograft was significantly upregulated in the *MT1JP* overexpression group compared with NC group. **f** The xenografts were H&E stained and expression of ki67 were measured by immunohistochemistry

BGC-823 cells with *MT1JP* overexpression or negative control were subcutaneously injected into the back flank of nude mice. An effective higher expression level of *MT1JP* was observed in *MT1JP* overexpression group than that in negative control group. In line with in vitro analysis, the results indicated that the volume and weight of xenograft tumor were significantly lower in *MT1JP* overexpression group as compared with negative control group (Fig. 3a-e). Furthermore, H&E and immunohistochemistry for Ki67 was performed to detect the expression of Ki67, and results showed that *MT1JP* overexpression resulted in a substantial reduce of Ki67 protein expression (Fig. 3f). We also detected the expression of apoptosis mediators by western blot. The results indicated that overepresion of *MT1JP* was observedcorrelated with the increased cleaved caspase 3 and cleaved caspase 9 (Additional file 1: Figure S2).

### LncRNA *MT1JP* played a ceRNA role in regulating FBXW7 expression by binding to miR-92a-3p

**Fig. 4:**
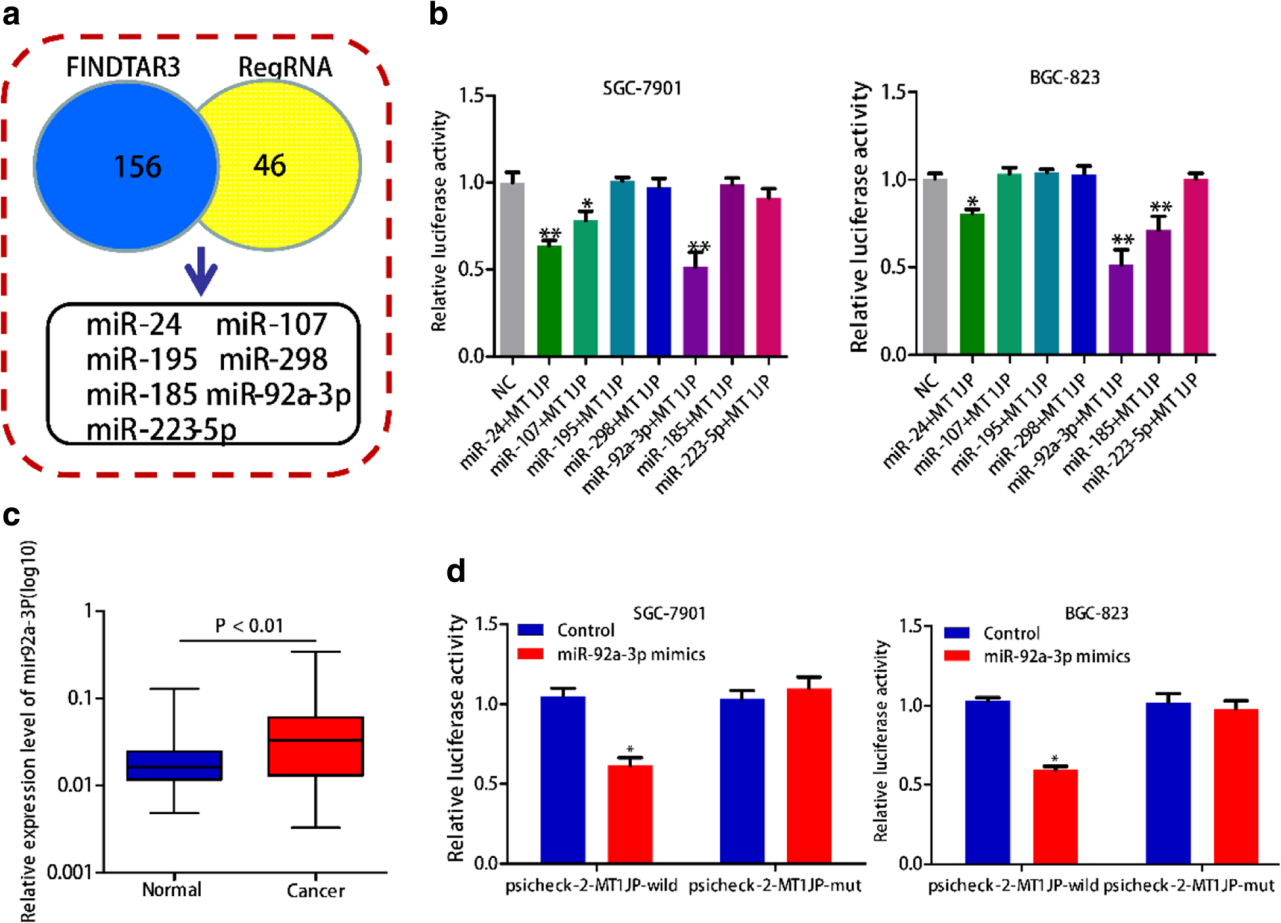
LncRNA *MT1JP* interacts with miR-92a-3p. **a** FINDTAR3 and RegRNA databases consistently predicted seven miRNAs interacted with lncRNA *MT1JP*. **b** Relative luciferase activity from SGC-7901 and BGC-823 cells co-transfected with *MT1JP* reporter plasmid and the candidate miRNAs. **c** The expression of miRNA92a-3p was significantly up-regulated in GC tissues compared with adjacent normal tissues. **d** miR-92a-3p mimic prominently reduced luciferase activity in *MT1JP*-wild not in *MT1JP*-mut

**Fig. 5:**
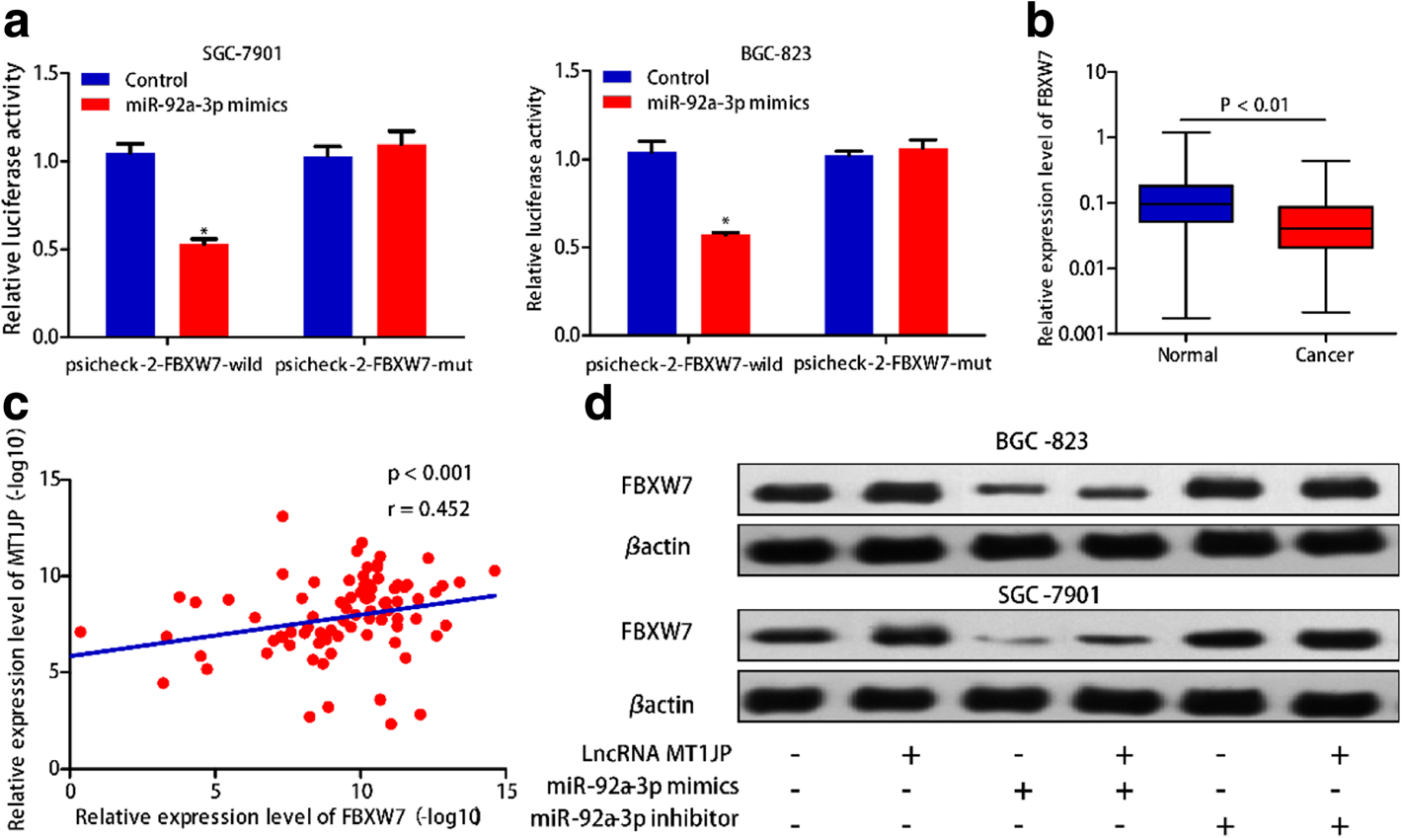
LncRNA *MT1JP* regulate FBXW7 expression by sponging miR-92a-3p. **a** miR-92a-3p significantly reduced luciferase activity in FBXW7-wild not in FBXW7-mut. **b** Compared with adjacent normal tissues, FBXW7 was remarkably upregulated in GC tissue. **c** Spearman-Pearson correlation between lncRNA *MT1JP* and *FBXW7* expression in GC tissues (Spearman *r* = 0.452, *P* < 0.001). **d** Western blotting analysis of FBXW7 in BGC-823 and SGC-7901 cells with overexpression of lncRNA *MT1JP* and/or miR-92a-3p mimics and/or miR-92a-3p inhibitor

The coding capability prediction of *MT1JP* was determined by online tool CPAT, and the result indicated that *MT1JP* had no apparent protein-coding ability (Additional file 1: Figure S3A). We next conducted nuclear-cytoplasmic fractionation to evaluate the lncRNA*MT1JP* subcellular localization, and found that lncRNA *MT1JP* was primarily detected in the cytoplasm of SGC-7901 and BGC-823 cells (Additional file 1: Figure S3B). To identify the potential miRNA targets of lncRNA *MT1JP*, in silico analysis was performed by using both FINDTAR3 (http://bio.sz.tsinghua.edu.cn/) and RegRNA (http://regrna.mbc.nctu.edu.tw/index1.php) databases, and jointly predicted that seven miRNAs (miR-24, miR-107, miR-195, miR-298, miR-185, miR-92a-3p and miR-223-5p) may act as biological targets of lncRNA *MT1JP* (Fig. 4a). We then conducted luciferase reporter assay to validate the binding of candidate miRNAs with lncRNA *MT1JP*, and selected miR-92a-3p with the mostly decreased luciferase activity into the further study (Fig. 4b). The expression levels of miR-92a-3p was significantly higher in GC tissues than in adjacent normal tissues (*P* < 0.01, Fig. 4c). In addition, miR-92a-3p mimic prominently decreased luciferase activity in *MT1JP*-wild not in *MT1JP*-mut in both SGC-7901 and BGC-823 cell lines, which indicated that miR-92a-3p is a target of lncRNA *MT1JP* (Fig. 4d). Further prediction of target genes of miR-92a-3p was performed by TargetScan (http://www.targetscan.org/vert_71/) and microRNA.org (http://www.microrna.org/microrna/home.do), and FBXW7 was selected as the target gene of miR-92a-3p for it acquired consistently highest score in the prediction analysis of both softwares. Luciferase reporter results showed that remarkably reduced luciferase activity was observed in FBXW7-wild not in FBXW7-mut (Fig. 5a). We used RT-PCR to assess the RNA expression of miR-92a-3p and FBXW7 in cells overexpressed MT1JP. The results indicated that the RNA level of FBXW7 significantly increased while no significant change in miR-92a-3p (Additional file 1: Figure S5). Moreover, overepresion of *MT1JP* markedly declined luciferase activity in miR-92a-3p-wild not in miR-92a-3p-mutant in both SGC-7901 and BGC-823 cell lines (Additional file 1: Figure S4A). Additionally, we detected the expression of miR-92a-3p correlate with MT1JP and FBXW7 in clinical samples. The results showed miR-92a-3p reversely correlate with FBXW7, but not correlate with MT1JP (Additional file 1: Figure S6).

We also found significantly lower expression of FBXW7 in GC tissues, as compared with corresponding normal tissues (*P* < 0.01, Fig. 5b). Moreover, a positive correlation between *MT1JP* and FBXW7 expression was detected in GC tissues (*r* = 0.452, *P* < 0.001, Fig. 5c). The western blotting and RT-PCR analysis results showed that miR-92a-3p mimic reduced the expression of FBXW7, whereas lncRNA *MT1JP* overexpression substantially abolished this effect. Additionally, lncRNA *MT1JP* had no up-regulated effect on FBXW7 with miR-92a-3p inhibitor (Fig. 5d) (Additional file 1: Figure S4B).

### MiR-92a-3p mimics and si-*FBXW7* reversed the suppression function of lncRNA *MT1JP* in vitro

**Fig. 6:**
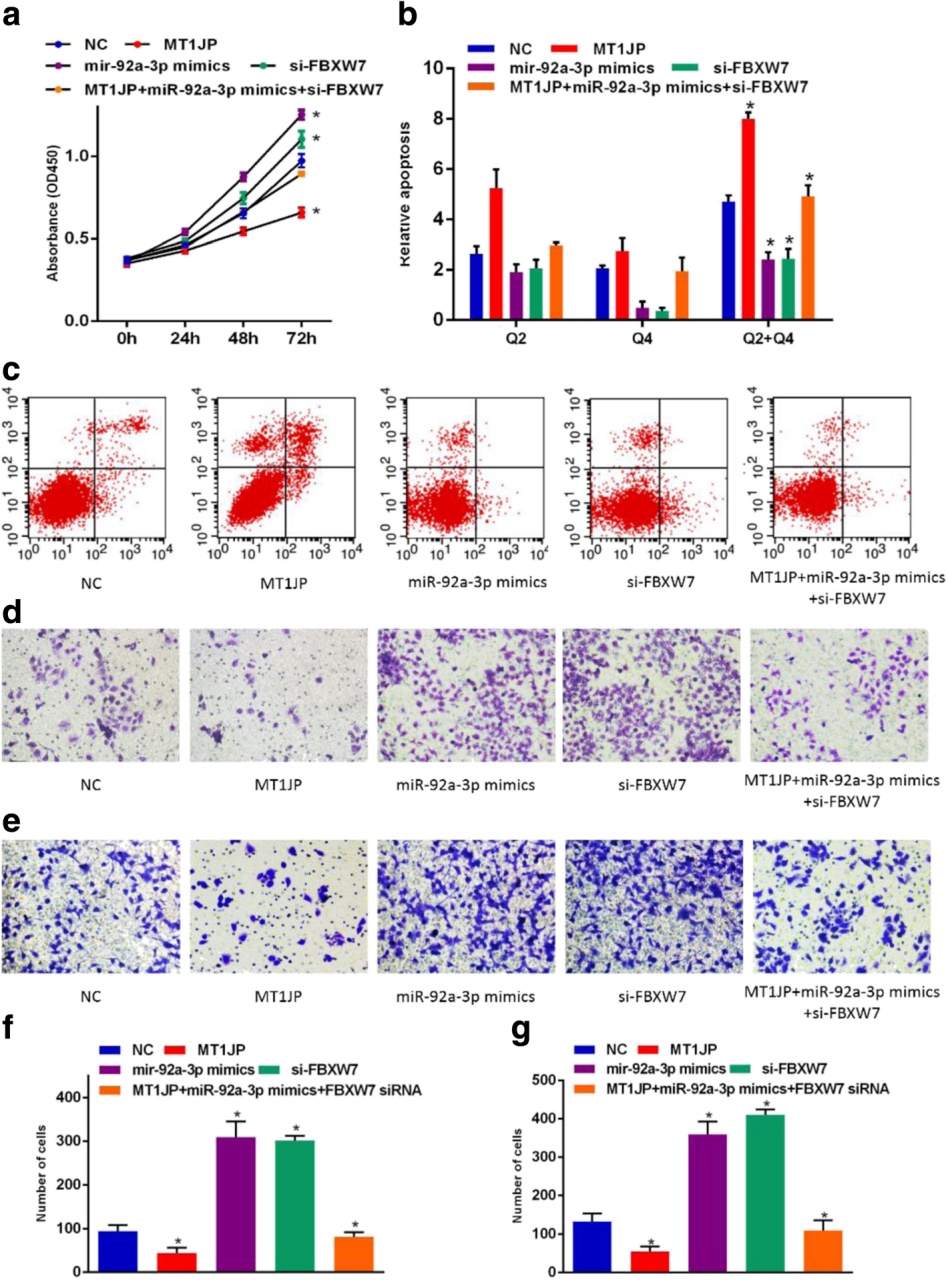
MiR-92a-3p mimics and si-*FBXW7* reversed the suppression function of lncRNA *MT1JP* in vitro. **a** Effect of MiR-92a-3p mimics, si-*FBXW7*, and lncRNA *MT1JP* overexpression on proliferation of GC cell line. **b**, **c** Effect of MiR-92a-3p mimics, si-*FBXW7*, and lncRNA *MT1JP* overexpression on apoptosis of GC cell line. **d**-**e** Effect of MiR-92a-3p mimics, si-*FBXW7*, and lncRNA *MT1JP* on migration and invasion of GC cell. Effect of miR-92a-3p mimics, si-FBXW7, and lncRNA *MT1JP* on proliferation of SGC7901 (**f**) and MGC-823 (**g**)

Since lncRNA *MT1JP* played a ceRNA role in regulating *FBXW7* expression by binding to miR-92a-3p, we performed rescue assays to validate whether miR-92a-3p and *FBXW7* were involved in the lncRNA *MT1JP*-mediated inhibition proliferation, migration and invasion and promoting apoptosis in GC cells. To determine the effects of miR-92a-3p and *FBXW7* in GC cell phenotypes, BGC-823 cell line was transfected with miR-92a-3p mimics and si-*FBXW7*, respectively. For rescue experiment, lncRNA *MT1JP* overexpression vector, miR-92a-3p mimics and si-*FBXW7* were co-transfected into BGC-823 cell. Intriguingly, both miR-92a-3p mimics and si-*FBXW7* remarkably promoted cell prolifereation, migration and invasion, and decreased cell apoptosis, respectively (Fig. 6). Co-transfection group (lncRNA *MT1JP* overexpression vector, miR-92a-3p mimics and si-*FBXW7*) could reversed the decrease on cell proliferation caused by lncRNA *MT1JP* overexpression (Fig. 6a). Moreover, flow cytometry analysis revealed that miR-92a-3p mimics and si-*FBXW7* significantly suppress apoptotic cell rate caused by lncRNA *MT1JP* overexpression (Fig. 6b-c). Cell migration and invasion experiments revealed that miR-92a-3p mimics and si-*FBXW7* prominently reversed the effects of lncRNA *MT1JP* overexpression (Fig. 6d-f).

## Discussion

There has been wide consensus that lncRNA play a key role in a variety of biological process, and aberrant expression or function of lncRNAs are commonly observed in a variety of cancers, including GC [2, 20]. Recently, lncRNA microarray was widely performed to investigate the lncRNA expression signatures, and identified significantly altered lncRNAs in cancers. In this study, we applied lncRNA microarray and integrated a GEO dataset to depict the lncRNA profiles, and found that *MT1JP* was the most significantly deregulated lncRNA with the largest Fold change. In the further validation study, the results showed that lncRNA *MT1JP* was significantly down-regulated in GC tissues compared with adjacent normal tissues. In line with the our results, a previous study applied an mRNA/lncRNA microarray in 76 pairs of tumor and normal tissue sample from live, colon, lung and gastric cancer, and revealed that lncRNA *MT1JP* had remarkably lower expression in all tumor samples [21]. Furthermore, our study found that expression of lncRNA *MT1JP* was prominently associated with lymph node metastasis and advance stage, suggesting a clinic pathological role of lncRNA *MT1JP* in GC. We also detected the involvement of miR-92 and FBXW7 in disease stage and progression. The results showed miR-92 and FBXW7 were associated with the TNM stage (Additional file 1: Figure S7 and S8). In addition, log-rank results demonstrated that GC patients with higher expression of lncRNA *MT1JP* had a well prognosis. These findings suggested that lncRNA *MT1JP* may act as a promising prognosis biomarker for GC. Additionally, we employed the ROC curve to calculate the prognostic significance of combination of MT1JP and miR-92a. The result indicated combination of MT1JP and miR-92a could improve the predictive value for prognosis of GC patient (Additional file 1: Figure S9).

To further explore the effect of *MT1JP* on cell phenotypes, *MT1JP* overexpression vector was constructed and transfected into SGC-7901and BGC-823 cells. Compared with negative control, lncRNA *MT1JP* overexpression significantly inhibited cell proliferation, migration and invasion and promoted cell apoptosis in both SGC-7901 cell and BGC-823 cell. Consistent with these findings in vivo, animal experimental study also confirmed that lncRNA *MT1JP* overexpreesion inhibited tumor growth and metastasis. Intriguing, Liu et al. reported that knockdown of *MT1JP* increased cell proliferation, migration and invasion while suppressed cell apoptosis in live cell line [21]. These results suggest a tumor-suppressor role of lncRNA *MT1JP* in GC and highlight the need for further study of the molecular mechanism of *MT1JP* involving in GC.

Nuclear-cytoplasmic fractionation were conducted to assess the subcellular localization and showed that majority of lncRNA *MT1JP* were located in cytoplasmic.

Emerging evidence support the ceRNA hypothesis, and indicated that ceRNA regulation implicated in the carcinogenesis of GC [22, 23]. It has been reported that lncRNA HOTAIR may act as a ceRNA to spong miR-331-3p and regulate the expression of HER2 in GC [24]. Therefore, we speculated that lncRNA may function as a ceRNA, participate in carcinogenesis of GC. Bioinformatics analysis and luciferase reporter assay confirmed that lncRNA *MT1JP* is a target of miR-92a-3p. Furthermore, our functional analysis also revealed that FBXW7 is a direct target of miR-92a-3p.

Given that both lncRNA *MT1JP* and FBXW7 interact with miR-92a-3p, suggesting that lncRNA *MT1JP* may regulate FBXW7 expression by competing bind to miR-92a-3p. In this study, the correlation analysis results showed that a significantly positive correlation between lncRNA *MT1JP* and FBXW7 expression was observed in GC tissues. Moreover, western blotting analysis also showed that miR-92a-3p mimic suppressed the expression of FBXW7, whereas lncRNA *MT1JP* overexpression substantially abolished this effect. Besides, although *MT1JP* was overexpressed, FBXW7 expression was not remarkably altered when knocked down miR-92a-3p. These results supported that lncRNA *MT1JP* involved in post-transcriptional regulation of FBXW7 by sponging miR-92a-3p.

Mounting evidence indicated that miR-92a-3p was often dysregulated in GC, and expression of miR-92a was remarkably related to the development of GC and had a prognosis value for GC [25, 26]. In this study, compared with negative control, miR-92a-3p significantly increased cell proliferation, migration and invasion and inhibited cell apoptosis in BGC-823 cell. Intriguing, in line with our study, miR-92a-3p was reported to promote tumor growth in multiple tumors by targeting FBXW7 [27–29]. FBXW7 is a member of SCF (complex of SKP1, CUL1 and F-box protein)-type ubiquitin ligase complex, which regulate target protein ubiquitination and degradation. The substrates of FBXW7 include several broadly investigated oncoproteins, such as MYC, cyclin E, mTOR and Notch. It is well known that FBXW7 is a tumor suppressor and its down-regulation was displayed in numerous human malignancies, including GC [30–32]. FBXW7 was reported to regulate tumor apoptosis, growth arrest and epithelial-to-mesenchymal transition in GC, and it also played an important role in drug resistance [33, 34]. The E-cadherin and MT1JP acted as the key proteins in the epithelial-to-mesenchymal transition. We use The Cancer Genome Atlas(TCGA) to explore the association between the FSP1and E-cadherin and MT1JP. The results indicated the expression of FSP1and E-cadherin were significantly associated with the expression of *MT1JP* **(**Additional file 1: Figure S10), which suggested *MT1JP* may play important role in epithelial-to-mesenchymal transition. In the present study, we have also shown that lncRNA *MT1JP* regulate FBXW7 expression by competition for miR-92a-3p binding. Up-regulated lncRNA *MT1JP* may lead to increase the expression of FBXW7, and consequently inhibited cell proliferation and promoted cell apoptosis in GC. These changes in GC cells could be reversed by miR-92a mimics and si-FBXW7 vector, according to rescue experiments in vitro.

## Conclusions

In summary, we identified *MT1JP*, a significantly down-regulated lncRNA, was associated with advance stage of GC, and GC patients with higher expression of *MT1JP* had a poorer survival, suggesting a promising prognostic role of *MT1JP* in GC. Functional analysis suggested that lncRNA *MT1JP* overexpression inhibited cell proliferation, migration, invasion and promoted cell apoptosis. Rescue assays suggested up-regulated miR-92a-3p and down-regulated FBXW7 reversed cell phenotypes caused by lncRNA *MT1JP*. Our study provided new insight into the post-transcriptional regulation mechanism of lncRNA *MT1JP* involved in the development of GC.

## Additional file

Additional file 1: **Figure S1-S10** (Online). **Table S1.** The top 10 significantly upregulated and downregulated lncRNAs identified by Arraystar Human lncRNA/mRNA chip. **Table S2.** The prime sequences of target genes used in real-time PCR. (DOCX 1257 kb)

## Abbreviations

ceRNA: competing endogenous RNA
GC: Gastric cancer
GEO: Gene Expression Omnibus
lncRNA: long noncoding RNA
MT1JP: Metallothionein 1 J, pseudogene
qRT-PCR: Quantitative reverse transcription polymerase chain reaction
TCGA: The Cancer Genome Atlas
