# Supplementary material for: LncRNA MT1JP functions as a ceRNA in regulating FBXW7 through competitively binding to miR-92a-3p in gastric cancer
Source: Mol Cancer. 2018 May 2;17:87. doi: 10.1186/s12943-018-0829-6 (PMC5930724; doi:10.1186/s12943-018-0829-6)

**LncRNA *MT1JP* functions as a ceRNA in regulating FBXW7 through competitively binding to miR-92a-3p in gastric cancer**

Gang Zhang^1,2,3#^, Shuwei Li^2,3#^, Jiafei Lu^2,3#^, Yuqiu Ge^2,3^, Qiaoyan Wang^2,3^, Gaoxiang Ma^2,3^, Qinghong Zhao^4^ , Dongdong Wu^4^, Weida Gong^5^, Mulong Du^2,3^, Haiyan Chu^2,3^, Meilin Wang^2,3^, Aihua Zhang^2,3^* and Zhengdong Zhang^2,3^*

^1^Department of Neurology, Children's Hospital of Nanjing Medical University, Nanjing, China.

^2^Department of Environmental Genomics, Jiangsu Key Laboratory of Cancer Biomarkers, Prevention and Treatment, Collaborative Innovation Center for Cancer Personalized Medicine, Nanjing Medical University, Nanjing, China.

^3^Department of Genetic Toxicology, The Key Laboratory of Modern Toxicology of Ministry of Education, School of Public Health, Nanjing Medical University, Nanjing, China.

^4^Department of General Surgery, The Second Affiliated Hospital of Nanjing Medical University, Nanjing, China

^5^Department of General Surgery, Yixing Cancer Hospital, Yixing, China

**^6^**Key Laboratory of Environmental Pollution Monitoring and Disease Control, Ministry of Education, Guizhou Medical University, Guiyang, Guizhou, China

^#^ These authors contribute to this work equally.

***Correspondence to:** Zhengdong Zhang, Department of Environmental Genomics, School of Public Health, Nanjing Medical University, 101 Longmian Avenue, Jiangning District, Nanjing 211166, China; Tel: +86-25-8686-8423; Fax:+86-25-8686-8499; Zhengdong Zhang, Email: drzdzhang@gmail.com or Aihua Zhang, Key Laboratory of Environmental Pollution Monitoring and Disease Control, Ministry of Education, Guizhou Medical University, Guiyang, Guizhou, China , 550025, Email:aihuagzykd@163.com

**Running title: lncRNA *MT1JP* and gastric cancer**

**Key words: lncRNA; *MT1JP*; gastric cancer; ceRNA; prognosis**

**Abbreviations:** GC, gastric cancer; lncRNA, long noncoding RNA; ceRNA, competing endogenous RNA; MTIJP, metallothionein 1J, pseudogene; TCGA, The Cancer Genome Atlas; GEO, Gene Expression Omnibus; qRT-PCR, quantitative reverse transcription polymerase chain reaction

**Additional file 1: Figure S1. Expression of lncRNA *MT1JP* in human GC tissues and GC cell lines. (A)** LncRNA *MT1JP* was significantly lower in GC tissues than in adjacent normal tissues from TCGA database. **(B)** The expression level of lncRNA *MT1JP*was relatively lower in BGC-823 and SGC-7901 cells.

**Figure S2. Expression of apoptosis mediators in GC mice tumor tissues.** The cleaved caspase 3 and cleaved caspase 9 protein expression in tumor tissues by transfection overexpression lncRNA *MT1JP* vector or not were determined by western blot.

**Figure S3. The coding capability prdiction and subcellular localization of lncRNA *MT1JP*. (A)** LncRNA *MT1JP* was predicted to have no ability to code a protein (coding probability< 0.364). **(B)** Majority of lncRNA *MT1JP* was found in the cytoplasm.

**Figure S4. LncRNA *MT1JP* alters FBXW7 expression by binding with miR-92a-3p. (A)**The overepresion of lncRNA *MT1JP* declined luciferase activity in miR-92a-3p-wild not in miR-92a-3p-mutant in both SGC-7901 and BGC-823 cell lines. **(B)** RT-PCR of FBXW7 in BGC-823 and SGC-7901 cells with overexpression of lncRNA *MT1JP* and/or miR-92a-3p mimics and/or miR-92a-3p inhibitor.

**Figure S5.** RT-PCR assessed the RNA expression of miR-92 and FBXW7 in cells overexpressed MT1JP. The RNA level of FBXW7 significantly increased while no significant change in miR-92.

**Figure S6.** The association between miR-92 and MT1JP and FBXW7 in clinical samples. The expression of miR-92 reversely correlate with FBXW7(A), but not correlate with MT1JP (B).

**Figure S7.** The expression of miR-92a in human GC tissues. The miR-92a was significant related with the TNM stage.

**Figure S8.** The expression of FBXW7 in human GC tissues. The FBXW7 was significant related with the TNM stage.

**Figure S9.** The ROC curve calculate the prognostic significance of combination of MT1JP, miR-92 and FBXW7. The AUC of MT1JP, miR-92 and FBXW7 were 0.60, 0.56, and 0.59, respectively. The combination of MT1JP and miR-92a could improve the predictive value for prognosis of GC patient(AUC=0.67).

**Figure S10.** The Cancer Genome Atlas(TCGA) was used to explore the association between the FSP1and E-cadherin and MT1JP. The results indicated the expression of FSP1and E-cadherin were significantly associated with the expression of MT1JP. The correlation coefficient were 0.23 and 0.19 for FSP1and E-cadherin, respectively.

**Table S1. The top 10 significantly upregulated and downregulated lncRNAs identified by Arraystar Human lncRNA/mRNA chip**

| Probename | Seqname | Relationship | FC | *P*value | FC | *P*value | Altered expression |
| --- | --- | --- | --- | --- | --- | --- | --- |
| ASHG19A3A054619 | *MT1JP* | intronic | 5.38 | 0.006 | 9.22 | 0.006 | downregualted |
| ASHG19A3A055309 | uc010vhg.1 | intergenic | 3.98 | 0.001 | 5.52 | 0.001 | downregualted |
| ASHG19A3A012995 | uc001hyk.1 | intergenic | 3.80 | 0.002 | 7.57 | 0.001 | downregualted |
| ASHG19A3A014064 | ENST000421314 | intergenic | 3.79 | 0.001 | 7.49 | 0.001 | downregualted |
| ASHG19A3A018253 | ENST000421424 | intergenic | 3.66 | 0.001 | 7.14 | 0.001 | downregualted |
| ASHG19A3A025603 | ENST000294916 | intergenic | 2.97 | 0.001 | 6.03 | 0.001 | downregualted |
| ASHG19A3A028263 | uc003jsf.3 | antisense | 2.80 | 0.035 | 6.98 | 0.001 | downregualted |
| ASHG19A3A051180 | BC047917 | intergenic | 2.75 | 0.040 | 7.36 | 0.001 | downregualted |
| ASHG19A3L000442 | AW179216 | intergenic | 2.68 | 0.049 | 4.79 | 0.001 | downregualted |
| ASHG19A3A054616 | MT1L | intergenic | 2.48 | 0.001 | 3.76 | 0.001 | downregualted |
| ASHG19A3A036942 | AK027294 | sense-overlapping | 12.50 | 0.003 | 2.08 | 0.002 | upregulated |
| ASHG19A3A022157 | uc003fxi.2 | sense-overlapping | 6.97 | 0.017 | 3.24 | 0.044 | upregulated |
| ASHG19A3A008651 | uc002iyl.2 | intergenic | 6.39 | 0.009 | 8.14 | 0.001 | upregulated |
| ASHG19A3A047643 | NR_003716 | Intergenic | 4.05 | 0.032 | 3.87 | 0.017 | upregulated |
| ASHG19A3A014186 | ENST000421567 | Intergenic | 3.28 | 0.010 | 2.31 | 0.005 | upregulated |
| ASHG19A3A020762 | ENST000445502 | Intergenic | 3.24 | 0.038 | 2.31 | 0.019 | upregulated |
| ASHG19A3A042941 | uc001jtm.2 | Naturalantisense | 3.12 | 0.025 | 2.31 | 0.019 | upregulated |
| ASHG19A3A040421 | uc004exm.2 | intergenic | 3.02 | 0.038 | 3.45 | 0.000 | upregulated |
| ASHG19A3A017977 | NR_027700 | sense-overlapping | 2.99 | 0.029 | 2.06 | 0.024 | upregulated |
| ASHG19A3A015404 | BC047034 | naturalantisense | 2.57 | 0.017 | 3.64 | 0.018 | upregulated |

**Table S2. The prime sequences of target genes used in real-time PCR**

| ID | Gene | Primer sequence |
| --- | --- | --- |
| 1 | *MT1JP* | F: 5'-CTCCTGCAAGAAGAGCTGC-3' |
|  |  | R: 5'-TGCAGCAAATGGCTCAGTA-3' |
| 2 | miR-92a-3p | F: 5'-CTCAACTGGTGTCGTGGAGTCGGCAATTCAGTTGATACAGGCCG-3' |
| 3 | FBXW7 | F: 5'-CGAACTCCAGTAGTATTGTGGACCT-3' |
|  |  | R: 5'-TTCTTTTCATTTTTGTTGTTTTTGTATAGA-3' |
| 4 | GAPDH | F: 5'-AAGGTGAAGGTCGGAGTCAAC-3' |
|  |  | R: 5'-GGGGTCATTGATGGCAACAATA-3' |
| 5 | U6 | F: 5'-AACGCTTCACGAATTTGCGT-3' |

**Supplementary Figure 1.**


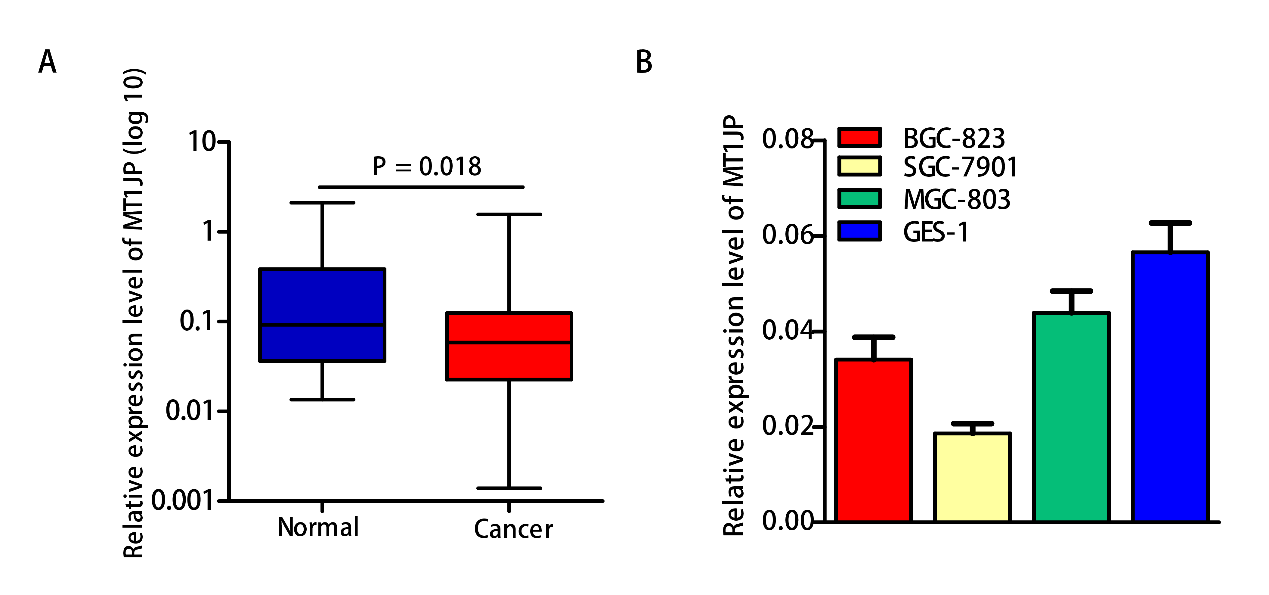


**Supplementary Figure 2.**

**
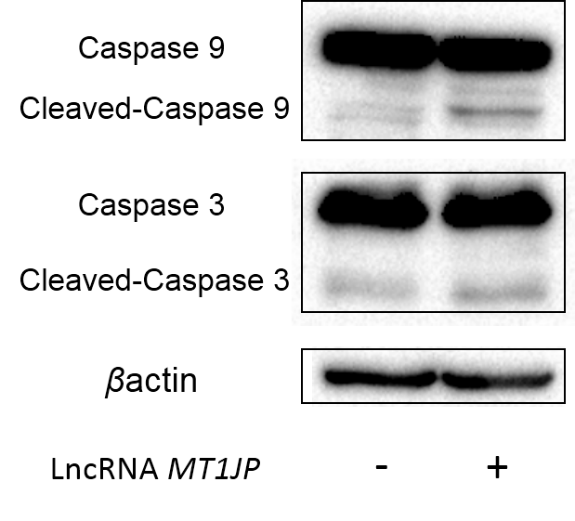
**

**Supplementary Figure 3.**


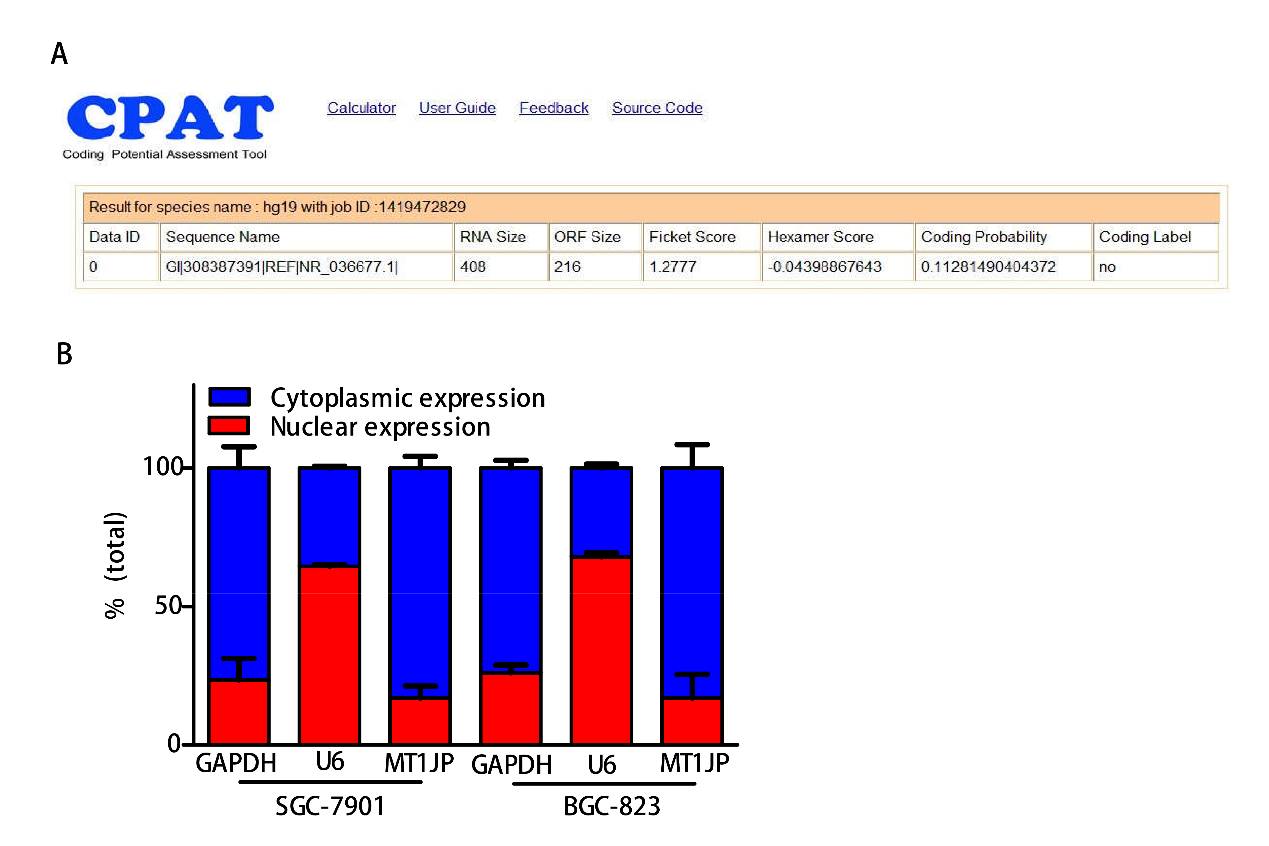


**Supplementary Figure 4.**

**

**

**Supplementary Figure 5.**


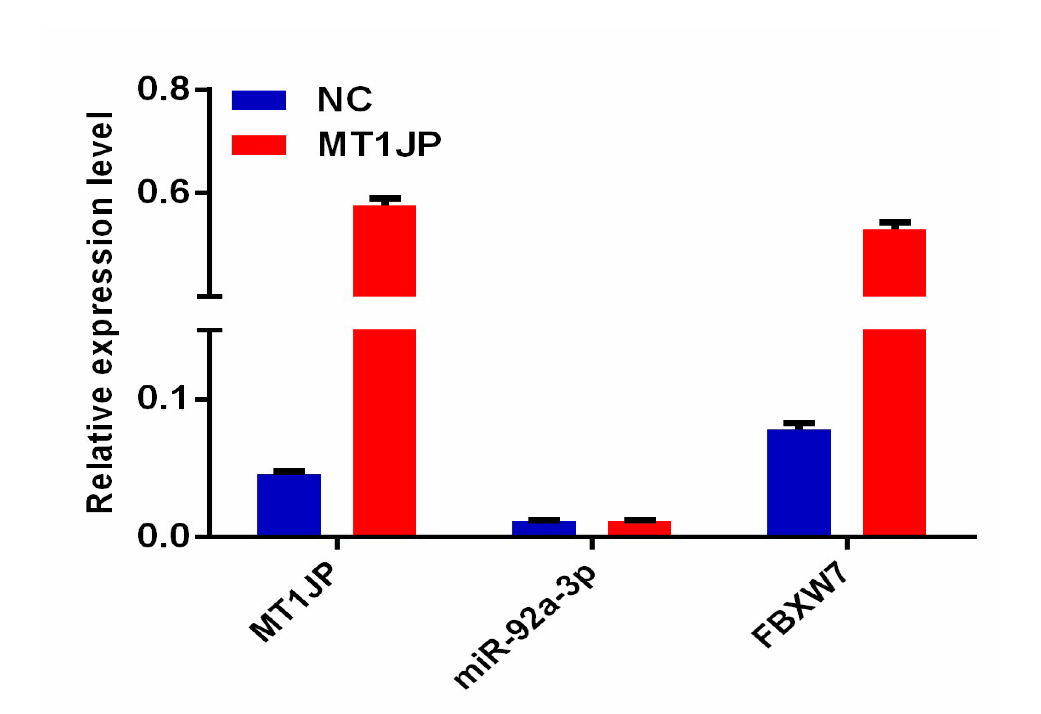


**Supplementary Figure 6.**


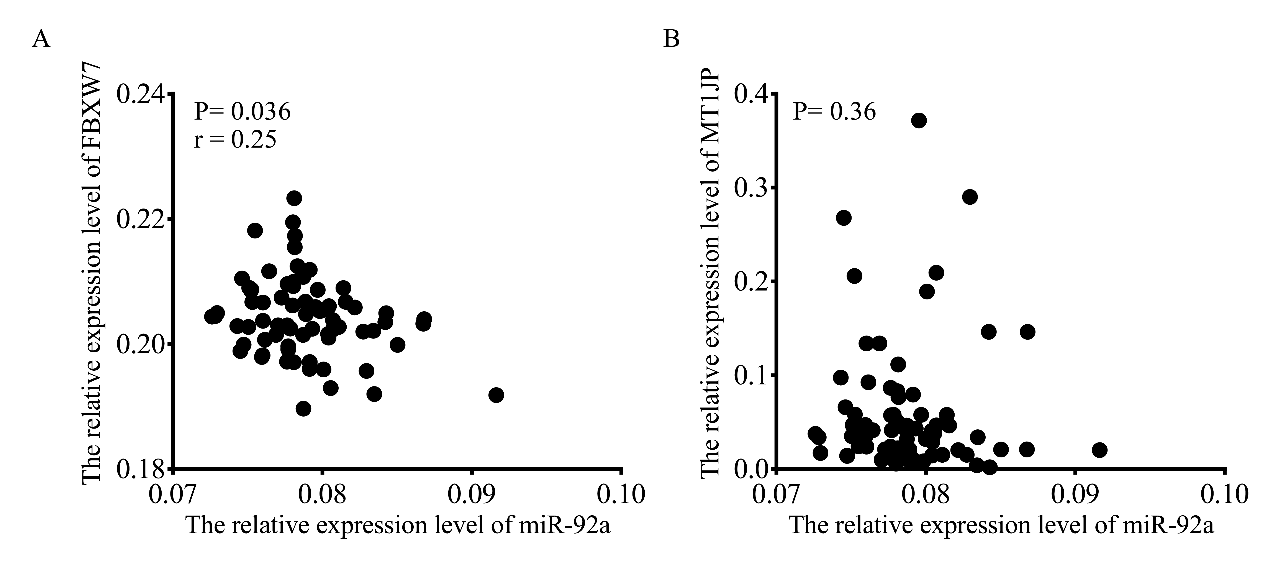


**Supplementary Figure 7.**


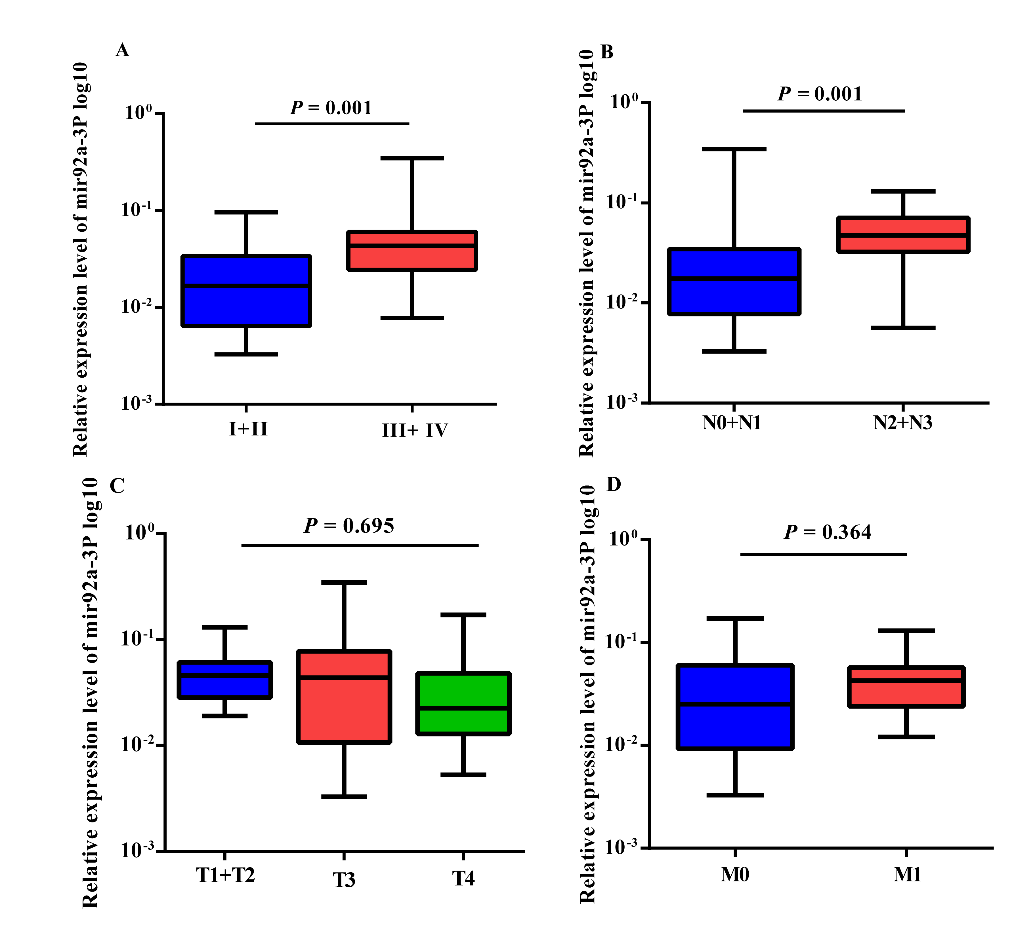


**Supplementary Figure 8**


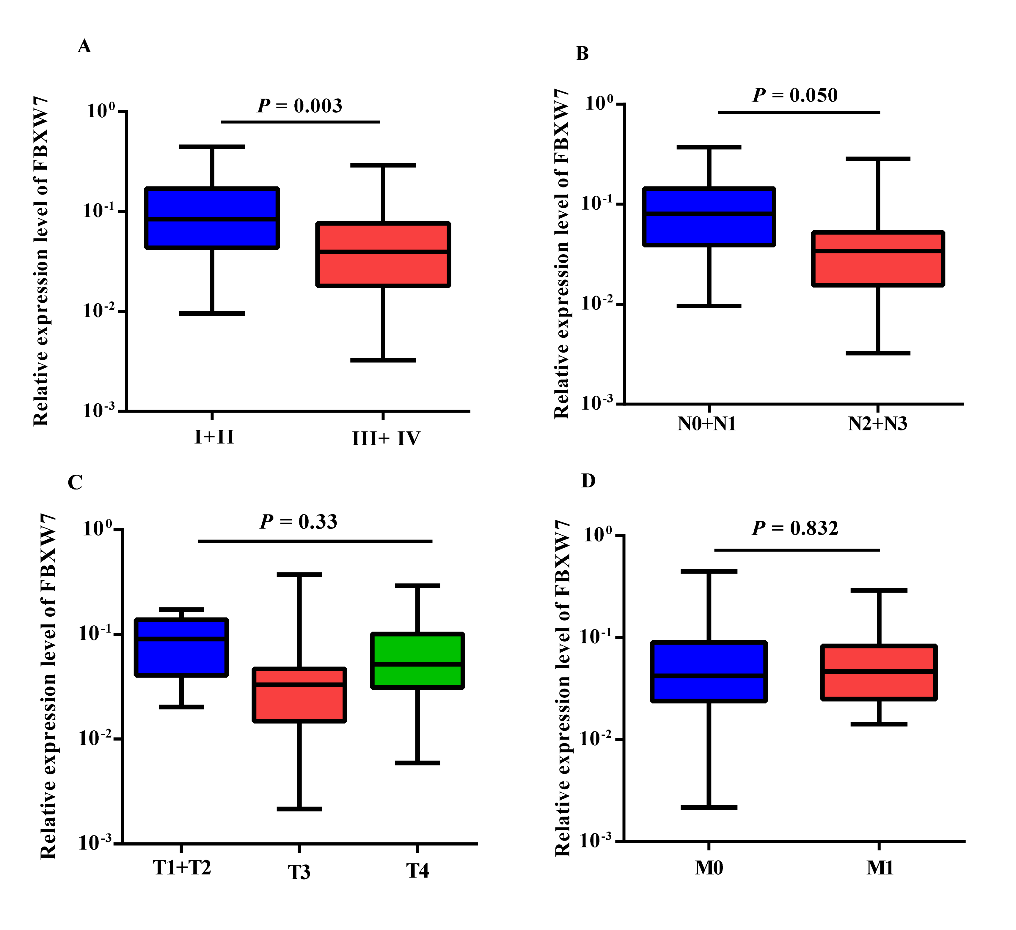


**Supplementary Figure 9**


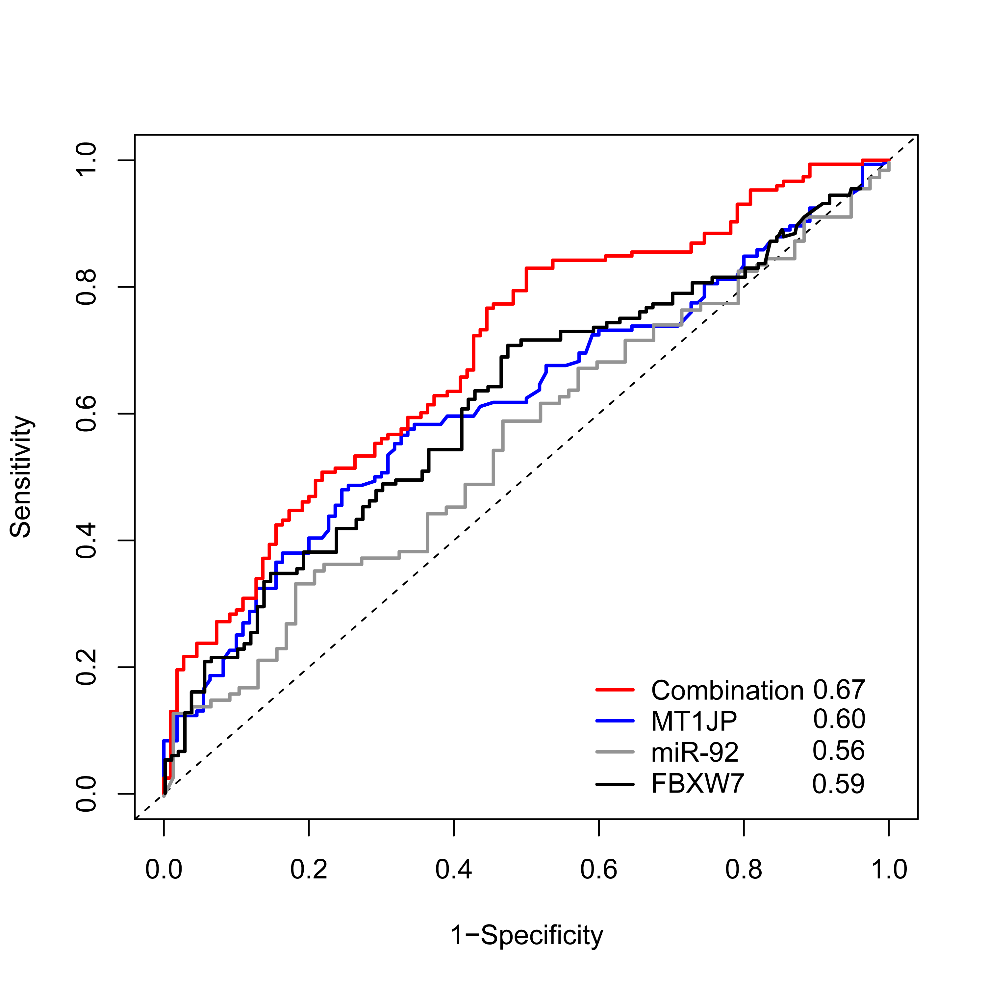


**Supplementary Figure 10**


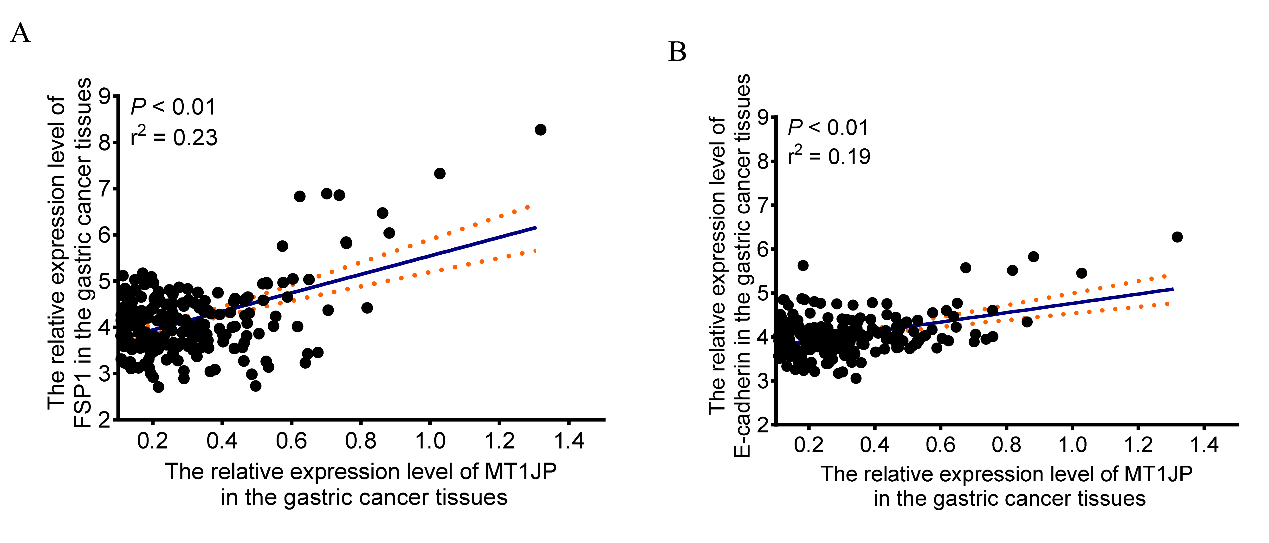

Supplement: Supplementary file 1 — Figure S1-S10 (Online). Table S1. The top 10 significantly upregulated and downregulated lncRNAs identified by Arraystar Human lncRNA/mRNA chip. Table S2. The prime sequences of target genes used in real-time PCR. (DOCX 1257 kb) [file 12943_2018_829_MOESM1_ESM.docx]
